# Application of high resolution T1 mapping with MOLLI (hrMOLLI) to differentiate patients with diffuse and regional myocardial disease from healthy subjects

**DOI:** 10.1186/1532-429X-14-S1-P225

**Published:** 2012-02-01

**Authors:** Ana Pastor, Zhong Chen, Tobias Voigt, Reza Razavi, Tobias Schaeffter, Eike Nagel, Valentina O Puntmann

**Affiliations:** 1 Cardiovascular Imaging, King's College London, London, UK; 2 Philips Research, London, UK

## Summary

Cardiac magnetic resonance (CMR) is increasingly applied as the first line investigation into the causes of myocardial dysfunction and remodelling. Although regional fibrosis is easily imaged on late gadolinium enhancement, characterisation of myocardial conditions with diffuse fibrosis remains challenging. Several studies proposed T1 mapping of gadolinium-enhanced myocardium supported by an inverse relationship between T1 relaxation times and amount of fibrotic tissue on histology. We performed high-resolution modified Look-Locker inversion recovery (hrMOLLI) imaging of native and postcontast myocardium in thirty-six subjects who underwent a routine CMR protocol for investigation of cardiomyopathy.

## Background

Several studies proposed T1 mapping of gadolinium-enhanced myocardium supported by an inverse relationship between T1 relaxation times and amount of fibrotic tissue on histology. To date no studies characterised the T1 values in non-enhanced myocardium in conditions with diffuse and regional fibrosis, nor evaluated its relation to post-contrast T1 values consistently in a clinical setting.

## Methods

We performed high-resolution modified Look-Locker inversion recovery (hrMOLLI) imaging of native and postcontrast myocardium in thirty-six subjects (age (mean±SD) 47 ± 7.4 years) who underwent a routine CMR protocol for investigation of cardiomyopathy (hypertrophic cardiomyopathy: n=6; longstanding hypertension: n=7, previous myocardial infarction: n=12). Eleven subjects referred for exclusion of arythmogenic right ventricular cardiomyopathy with normal CMR findings served as controls. Single breath-hold hrMOLLI was performed on a 3T clinical scanner in an equatorial short axis slice prior and at 10, 20 and 40 minutes following administration of 0.2 mmol/kg gadolinium-DTPA contrast. Imaging parameters were FOV 320x320; TR/TE 3.2/1.57 ms, flip angle 50°, interpolated voxel size 0.9x0.9x8mm, phase encoding steps n=166, trigger delay: 450 msec. T1 map values were obtained by placing the region of interest (ROI) within the septal myocardium. In patients with myocardial scar on late gadolinium enhancement, ROIs were derived from both remote and scarred areas pre-contrast and 20 minutes afterwards only. In addition to the native and postcontrast T1 values the ratio between postcontrast and precontrast values was calculated.

## Results

**Figure 1:**
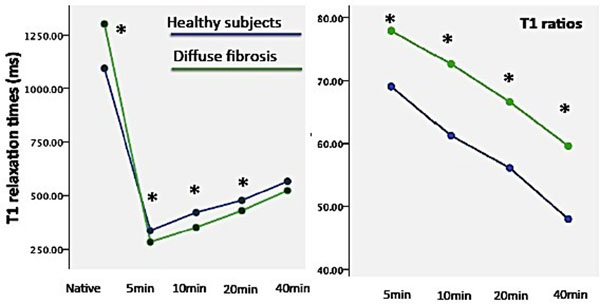
T1 relaxation times and ratios in healthy subjects (blue) and diffuse fibrosis (green). t-test, *indicates p<0.05.

**Figure 2:**
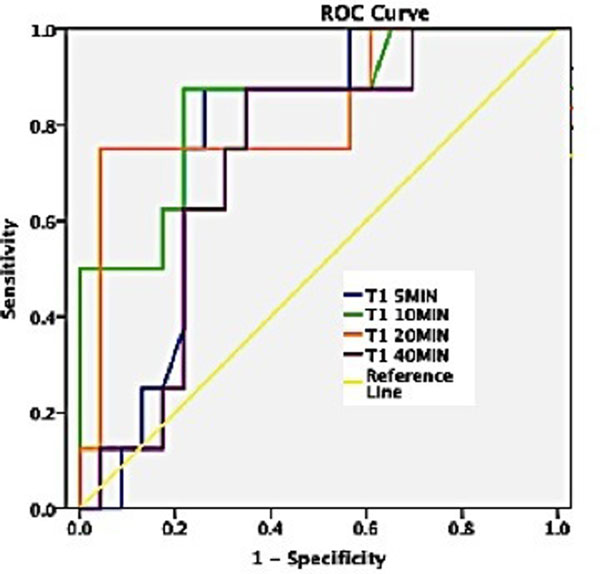
ROC analysis reveals a cut-off value of 320 ms 10min post injection provided the greatest distinction between healthy myocardium and diffuse fibrosis.

Native T1 relaxation times were shorter in healthy myocardium than in diffuse fibrosis or scarred areas (Figure 1), whereas T1 values in remote areas did not differ significantly from healthy myocardium. Postcontrast T1 values were significantly shorter in diffuse fibrosis at 5-20 minutes (p<0.05), but not at 40 minutes post injection. At 20 minutes, T1 values in scarred areas were significantly shorter than in healthy myocardium or diffuse fibrosis (p<0.01). The relative ratio between postcontrast and precontrast values showed significantly higher values for diffuse fibrosis in comparison to normal myocardium at all time-points (5-40min). For post contrast myocardium a cut-off value of 320 ms 10min post injection provided the greatest distinction between healthy myocardium and diffuse fibrosis (sensitivity 100%, specificity 83%, AUC=0.85, 95%CI: 0.68-1, p=0.004) (Figure 2). Distinction of normal and fibrotic tissue was significantly better with native than with contrast enhanced images (p<0.001).

## Conclusions

We demonstrate that T1 mapping of native and postcontrast myocardium can serve to differentiate between healthy myocardium and diffuse fibrosis with better accuracy than after contrast injection.

## Funding

NIHR British Research Centre.

